# Preparation and Characterization of PLGA Nanoparticles Containing Plasmid DNA Encoding Human IFN-lambda-1/IL-29

**Published:** 2019

**Authors:** Parisa Amir Kalvanagh, Masoumeh Ebtekar, Parviz Kokhaei, Hoorieh Soleimanjahi

**Affiliations:** a *Department of Immunology, Faculty of Medical Sciences, Tarbiat Modares University, Tehran, Iran.*; b *Department of Immunology, Semnan University of Medical Sciences, Semnan, Iran.*; c *Department of Virology, Faculty of Medical Sciences, Tarbiat Modares University, Tehran, Iran* *.*

**Keywords:** IFN-λ1, PLGA, Non-viral gene delivery, Therapy

## Abstract

During the 15 years since the discovery of type III human interferons [IFN-λ1(IL-29), IFN-λ2(IL-28A), and IFN-λ3(IL-28B)], numerous biological properties such as anticancer, antiviral, and immunomodulatory activities of this new IFN family have been investigated. Several studies have shown that the encapsulation of pcDNA with PLGA nanoparticles (NPs) protects them against DNase enzyme action and increases the efficiency of gene delivery to the cells. The purpose of this study was to encapsulate pcDNA encoding IFN-λ1 (pIFN-λ1) with a simple and cost-effective method using PLGA NPs. The pIFN-λ1-loaded PLGA NPs were produced by a double-emulsion-solvent evaporation method and characterized in terms of size, size distribution, and zeta potential by DLS and morphologically by SEM and TEM. The bioactivity of NPs was also examined by fluorescent microscopy. The results showed that IFN-λ1 expressed by HEK293T cells could protect HepC-2 cells from the cytopathic effects of EMCV. The NPs were spherical in shape with a mean diameter of 380 ± 3 nm, a zeta potential of −3.3 ± 7.6 mV, an encapsulation efficiency of 75 ± 5%, and a loading capacity of 0.83 ± 0.06. The NPs were also bioactive and easily engulfed by RAW264.7 cells. The pIFN-λ1 could be sustainably released from NPs. Due to the facility and affordability of encapsulation of pIFN-λ1 in the PLGA NPs proposed in this study and the advantages of encapsulation by PLGA, it appeared rational to use pIFN-λ1-loaded NPs instead of naked pIFN-λ1 to determine other unexplained activities of this new cytokine or to use it as an alternative or adjunct to current IFN-α therapy.

## Introduction

 Interferons (IFNs) are the largest cytokine family, and they are classified into three categories: type I, type II, and the most recently discovered, type III IFNs (1). The human genome encodes four genes associated with type III IFNs. Three genes from this family, IFN-λ1 (IL-29), IFN-λ2 (IL-28A), and IFN- λ3 (IL-28B), were identified in 2003 (2, 3), and IFN-λ4 was introduced as a pseudogene at that time. Ten years later, it became clear that most humans have functional IFN-λ4, and an single-nucleotide polymorphism (SNP) leading to frameshift mutations in some populations ceases production of this interferon (4). Over the past 15 years since the identification of type III IFNs, numerous research studies have been conducted, and their results reflect their crucial role in animal models of cancer (5), autoimmune disease, (6) and viral infections (7). 

 Currently, IFN-α is used as an FDA-approved treatment for chronic hepatitis C virus (HCV) (8) and hepatitis B virus (HBV) infections (9). Although therapeutic effects of IFN-α have been proven, substantial adverse effects, including flu-like symptoms, lymphopenia (10, 11), and pulmonary arterial hypertension (PHA), (12) are still being reported by patients and clinicians. Targeting diseases using IFN-λ instead of or together with IFN-α to reduce the frequency of adverse events was an idea resulted from the observation that expression of the IFN-λ receptor (IFN-λR1) was limited to only a few cell types, in contrast with the interferon alpha receptor, which is expressed on the surface of all cells (13). In a randomized, blinded, actively controlled, multicenter Phase IIb variable-dose study, IFN-λ treatment led to greater HCV suppression at week 12 and equal inhibition at week 24 in comparison with IFN-α. However, in patients receiving IFN- λ, neutropenia and thrombocytopenia were rarely observed (14). In addition, numerous clinical studies on the human genome have demonstrated a significant correlation between SNPs in lambda interferon genes and their antiviral activity. For example, SNP rs12979860 was associated with the recurrence rate of HSV-1 (15), the response rate to IFN therapy in patients with HBV (16) and spontaneous clearance of HCV (17). Therefore, IFN-λ might be an alternative to current IFN-α therapy. 

 One of the common challenges associated with therapeutic cytokines is their short half-life (18). The use of gene delivery can increase the half-life and subsequently reduce the side effects, toxicity, and dosage of the drug. Moreover, *in-vivo *expression ensures that the protein more closely resembles the normal eukaryotic structure with accompanying post-translational modifications. However, DNA administered by intravascular injection is rapidly degraded by circulating nucleases, and the naked gene struggles to enter the cell (19). It therefore seems rational to obtain help from biological carriers to deal with these two major flaws in genetic material. Recently, poly-lactic-co-glycolic acid (PLGA) has attracted considerable attention due to its interesting properties of biodegradability and highly biocompatibility, and it has gained US Food Drug Administration and European Medicine Agency approval for drug delivery systems (20). The results of plasmid complementary DNA (pcDNA) encapsulation by PLGA have confirmed the excellent stability and integrity of encapsulated pcDNA, its protection against DNase I, and the sustained release of pcDNA *in-vitro *(21-24). The double-emulsion-solvent evaporation method, namely water-in-oil-in-water (w/o/w) is the most common method for encapsulation of water-soluble drugs such as pcDNA (25). Our aim in this study was to encapsulate pIFN-λ1 in PLGA NPs using this method in a simple and affordable way. 

## Experimental

*Materials*

 Poly (D, L-lactic-co-glycolic acid) (PLGA; lactic–glycolic acid ratio; 50:50) in a molecular mass range of 54-69 kDa (RG 505) were purchased from Sigma (Germany). Polyvinyl alcohol (PVA) with 98-99% degree of saponification, 25-30 mpas viscosity, the density of 1.25 g/cm^3^, and MW of 74.8 kDa were obtained from NipponGohsei (Japan). The recombinant IFN-λ1 (100 ng/mL) was purchased from Research and Development systems (USA). Quant-iT ™ PicoGreen dsDNA assay kit was purchased from Invitrogen (USA). Endofree plasmid Giga Kit was purchased from Qiagen (Germany). Human Embryonic Kidney cell line (HEK293T) were obtained from Pasteur Institute (Iran). Mouse macrophage cell line (RAW264.7) was obtained from Faculty of Veterinary Medicine of Tehran University (Iran). Cervix carcinoma cell line (Hep2-C) and Encephalomyocarditis virus (EMCV) were obtained from Food and Drug Administration (Iran). The expression vector of pcDH-GFP(pGFP) encoding green fluorescent protein was kindly gifted by Prof. H. Soleimanjahi. All other chemicals and solvents were common laboratory-grade reagents.

*Methods*

*Cloning and expression of pIFN-λ1*

 Cloning and expression of pcDNA3.1-IFN-λ1(pIFN-λ1) encoding interferon lambda-1 were done in our previous study (26). In summary, total RNA from human monocyte-derived dendritic cells stimulated with 100 ng/mL of LPS was extracted and cDNA synthesized. PCR was performed by specific primers of IFN-λ1 mRNA and cloned inside the PTZ57R /T vector and then subcloned into pcDNA3.1 + using KpnI and BamHI restriction endonucleases. After confirming the IFN-λ1 cDNA sequence by Nucleotide BlAST at NCBI, pIFN-λ1 transfected into HEK293T by the polyfect reagent, the expression of IFN-λ1 was confirmed by sandwich ELISA method (R&D system, USA). After transformation of pIFN-λ1 into *E.coli* (DH5a) using Cacl2 and cultivated in a high scale, purification of pIFN-λ1 was performed with Endofree plasmid Giga Kit according to the manufacturer's protocols. Similarly, pGFP purification was done.

*Antiviral assay of IFN-λ1glycoprotein*

 Before the encapsulation of pIFN-λ1 in PLGA, the biological activity of IFN- λ1 glycoprotein secreted in the supernatant of HEK293T cells transfected with the corresponding plasmid was analyzed by an antiviral assay as previously described (27). In brief, Hep-2C cells were cultured in 100 µL of EMEM (Eagle's Minimum Essential Medium) supplemented with 10% fetal bovine serum (FBS) and 1% (v/v) pen/strep in 96-well plates at a concentration of 1.5 × 10^5^ cells per well and incubated at 37 °C in 5% CO2 for 18 h. Two-fold serial dilution of IFN- λ1 secreted in the supernatant as well as recombinant IFN-λ1(100 ng/mL) were prepared using the culture medium and then transferred to the plate (Four wells were assigned to each dilution). After 24 h, the culture medium was aspirated and replaced by medium containing EMCV (3 × 10^7^ PFU/mL) in all wells other than cells control. The plate was incubated at 37 °C and when 10% of cells of virus control were alive, staining process was started. At first, the plate was washed 3-4 times with PBS and stained with naphthalene black for 25 min at room temperature. After fixation with 4% formalin acetate for 10 min, cells were washed again and then 150 μL of 0.1 M NaOH was added and shaken for 30 sec. Finally, the absorbance was read at 620 nm by the spectrophotometer.

*Preparation of pIFN-λ1-loaded PLGA NPs*

 The pIFN-λ1 was encapsulated in PLGA by the double-emulsion-solvent evaporation technique. For this purpose, 300 μg of pIFN-λ1 that was dissolved in 200 μL of Tris-EDTA buffer with pH 8.2 was added to 30 mg of PLGA which was dissolved in 1000 μL of chloroform and emulsified using microtip probe sonicator for 30 sec on the ice at 30% amplitude. This primary-water-in-oil emulsion was added dropwise to 6 mL of 1% (W/V) aqueous solution of PVA and sonicated for the 60s at 70% amplitude to form secondary-water-in-oil-in-water emulsion. In order to evaporate the chloroform, the secondary emulsion was agitated using a magnetic stirrer for ~18 h at 150 rpm. Then it was centrifuged for 10 min at 17000×g. The first supernatant was collected to calculate the non-encapsulated plasmids in the NPs. In order to remove PVA, pIFN-λ1-loaded NPs (F1 formulation) were washed twice with double-distilled water and resuspended in double distilled water. Finally, NPs were lyophilized for 24 h and stored at -70 °C for further investigation. Simultaneously, the same method was also used to produce empty NPs (F2 formulation) and the pGFP-loaded NPs. It is necessary to mention 300 μg of pGFP was also used.

*Encapsulation efficiency, loading capacity, and production yield estimation*

 The standard calibration curve of DNA concentration versus fluorescence intensity was drawn according to Quant-iT ™ PicoGreen® dsDNA assay kit, with and without of PVA, using a fluorimeter (Biotek, Senergr HT, Winooski, VT, USA) at wavelengths of 485/520 nm. The first supernatant after centrifuge was collected and diluted in TE buffer and the non-encapsulated pIFN-λ1s content was quantified using the calibration curve. This content was subtracted from the total amount of pIFN-λ1 used in the encapsulation process (initial pIFN-λ1) and was considered as encapsulated pIFN-λ1s.The principles of calculating all three concepts (Encapsulation efficiency, loading capacity, and production yield) were taken according to the equations used by Khalid Mohamed El-Say (28).

Encapsulation efficiency%=Weight of encapsulated pIFN­λ1 Weight of initial pIFN­λ1 ×100

Loading capacity%=Weight ofencapsulated pIFN­λ1 Weight of produced NPs×100

$\mathrm{Production yield}\left(\%\right)=\frac{\mathrm{Weight of produced NPs}}{\mathrm{Total weight of initial pIFN­\lambda 1 and PLGA}}\times 100$

*Morphological analysis of the NPs*

 In order to evaluate the morphology of pIFNλ1-loaded NPs and empty NPs, 1 mg of the NPs were added to 1 mL of distilled water and sonicated for 60 sec, and the resulting suspension was deposited on a scanning electron microscopy stub as a drop. After good drying at RT, NPs were coated with gold particles and their morphology were determined by a scanning electron microscope (SEM) (EM3200, KYKY, China) at an accelerating voltage of 26 kV with 10.0 KX magnitude. Three months after the storage of the lyophilized pIFNλ1-loaded NPs at -70 °C, they were re-examined by a transmission electron microscope (TEM) (ZIESS, EM900, PHILPS, Germany). For this purpose, the suspension of pIFNλ1-loaded NPs was added to a carbon grid. After drying at RT, the images were provided.

*Size, Size distribution and Zeta potential measurement of the NPs*

 To evaluate the size, size distribution and, zeta potential properties of developed NPs, 0.7 mg of each lyophilized NPs were resuspended in sterilized distilled water using a bath sonicator (Ultrasonic Cleaner Set, WUC-D10H, Korea) for 3 min at 20% power. The zeta potential and size distribution of resuspended NPs were documented by dynamic light scattering method (Malvern Zetasizer ZS, UK). Size distribution and Zeta potential of the pIFNλ1-loaded and empty NPs were simultaneously determined.

*In-vitro* *release pattern* *of pIFN-λ1 from the NPs*

 In order to find out the pattern of pIFN-λ1 release from the NPs, 1.5 mg of lyophilized pIFN-λ1-loaded NPs in 1 mL of PBS using bath sonicator were resuspended inside the DNase-RNase free tube at pH 7.2. The tubes were placed vertically in a shaker incubator (100 rpm) at 37 °C. At defined intervals (5 days in between), the samples were centrifuged for 5 min at 14000×g and supernatants were collected in new clean tubes. Immediately, the concentration of the released plasmids into the supernatant was calculated at 260 nm. The remaining supernatants were transferred to -20 °C and the NPs were resuspended in 1 mL of new PBS with pH 7.2 and placed back to a shaker incubator in the same condition. The concentration of all samples collected at -20 °C during 60 days was again measured with PicoGreen assay Kit. In fact, release pattern was investigated by two methods (spectrophotometry and fluorimetry). All release experiments were performed in triplicate.

**Table 1:** Average particle size, polydispersity index, and zeta potential of the NPs (n = 3)

**Formulation**	**Name**	**pIFN-λ1** **(µg)**	**Tris-EDTA buffer (μL)**	**PLGA** **(mg/mL)**	**PVA%** **(w/v)**	**Average Particle** **Size (nm) ± SD**	**Average Polydispersity** **Index ± SD**	**Average Zeta** **Potential (mV) ± SD**
F1	The pIFN-λ1-loaded NPs	300	200	30	1%	380 ± 3	0.26 ± 0.02	3.3 ± 7.6
F2	Empty NPs	0	200	30	1%	310 ± 4	0.24 ± 0.01	3.3 ± 7.9

**Table 2:** The percentage of viability RAW264.7 and HEK293T cell lines after four days treatment by empty NPs using Trypan blue viability test. Each concentration was performed in triplicate

	**The concentration of empty NPs**		
Cell lines	125 (μg/mL)	250 (μg /mL)	500 (μg /mL)
RAW264.7	97 ± 3%	96 ± 3%	95 ± 2%
HEK293T	95 ± 2%	94 ± 1%	93 ± 1%

**Table 3:** Encapsulation efficiency, loading capacity and production yield of the pIFN-λ1-loaded NPs estimated by the mentioned equations (n = 5)

**NPs**	**Encapsulation efficiency (%)**	**Loading capacity (%)**	**Production yield (%)**
The pIFN-λ1-loaded NPs	75 ± 5	0.83 ± 0.06	89 ± 0.5

**Figure 1:**
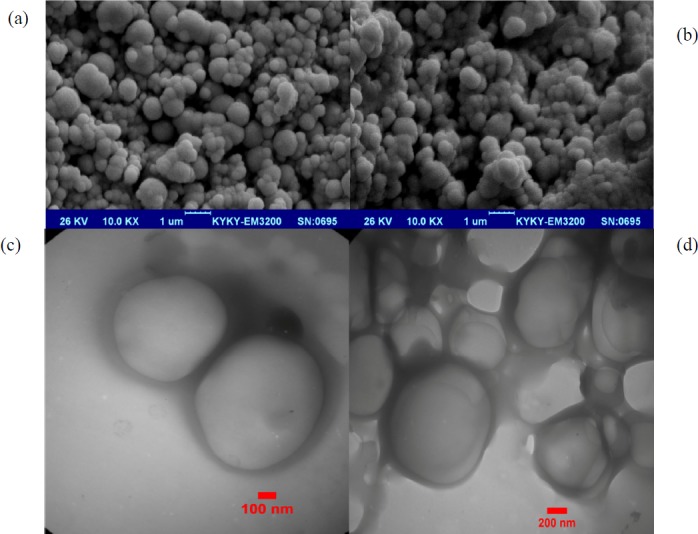
The SEM images of NPs prepared using the double-emulsion-solvent evaporation technique. (a) The pIFN-λ1-loaded NPs, and (b) empty NPs. (c and d) The TEM images of the pIFN-λ1-loaded NPs prepared using the double-emulsion-solvent evaporation technique after 3 months of storage at -70 °C

**Figure 2:**
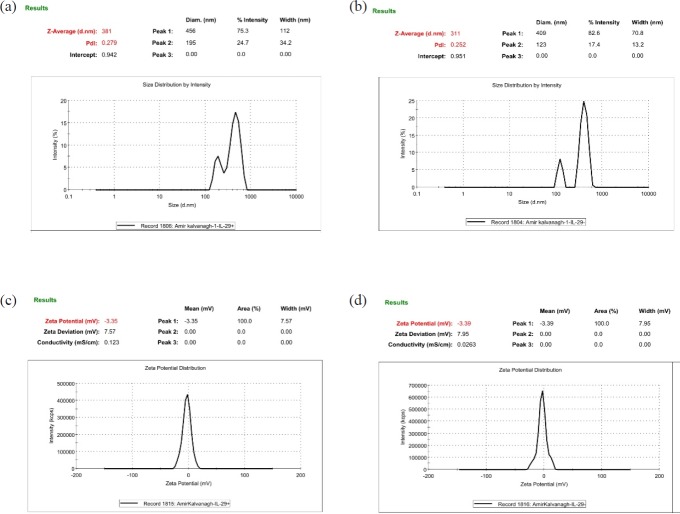
Comparing of the pIFN-λ-1-loaded NPs and empty NPs in terms of size, PdI, and zeta potential. All of the parameters are obtained by Zeta Sizer. (a and c) The NPs formulated with F1 formulation and (b and d) the NPs formulated with F2 formulation

**Figure 3:**
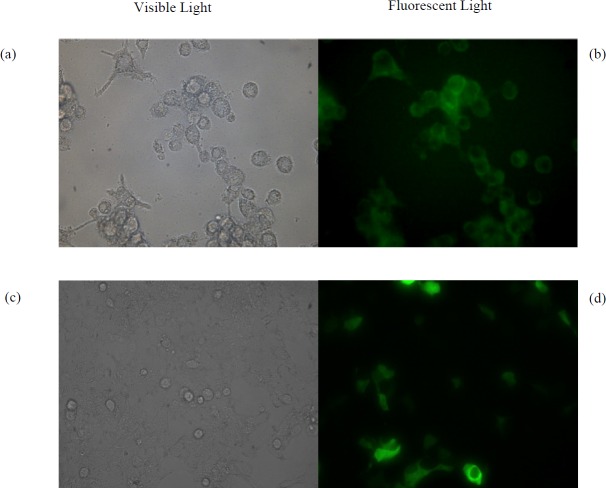
(a) Microscopic images of RAW264.7 cells under visible light and (b) fluorescent light treated by the pGFP- loaded NPs. (c) Microscopic images of HEK293T cells under visible light and (d) fluorescent light treated by free pGFP using calcium phosphate method

**Figure 4:**
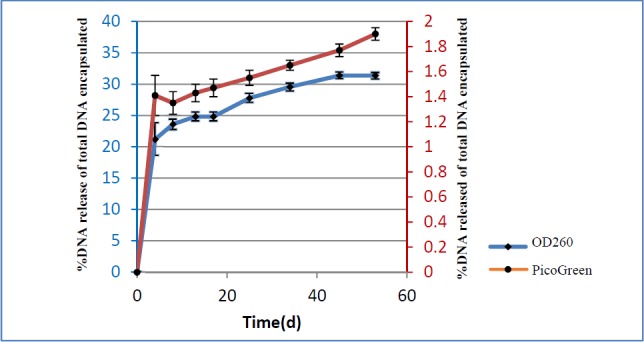
Release patterns of pIFN-λ1 from NPs were measured by Picogreen assay and UV absorbance at 260 nm

**Figure 5:**
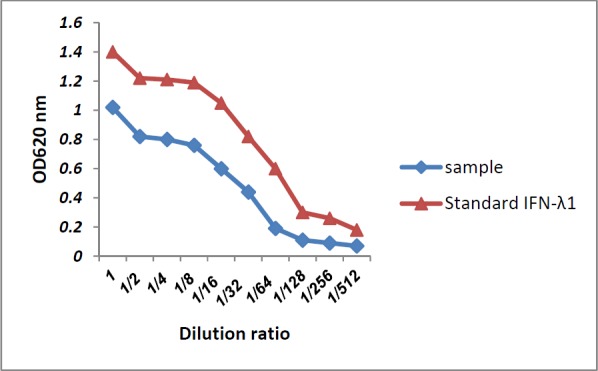
Antiviral activity of IFN-λ1/IL-29 secreted to the supernatant of HEK293T cells (sample) compared to recombinant IFN-λ1 using the CPER test

*Cellular uptake and bioactivity of the NPs*

 The HEK293T and RAW264.7 Cells were chosen as non-phagocytic and phagocytic cell lines, respectively. Both of them were grown in 2 mL of DMEM Medium supplemented with 5% fetal bovine serum (FBS) and 1% (v/v) pen/strep in 12-well plates at a concentration of 1.25 × 10^5^ cells per well for 22 h. Then the medium was aspirated, the cells were washed and 1 mL of fresh medium was added. After 2 h, cells were treated with 0.5 mg of suspended pGFP-loaded NPs by which the same method described for pIFN-λ1-loaded NPs were prepared. After18 h treatment, in order to remove NPs that were not uptaken by cells, 80% of the culture medium was gently collected and replaced with fresh medium. Expression of GFP was examined under fluorescence microscope four days after treatment of cells with NPs. As a positive control, pGFP transfected by calcium phosphate method in both cell lines and after two days examined under a fluorescence microscope. Untreated cells were used as negative control. In addition to these controls, both of cell lines were treated with three different concentration of empty NPs (125, 250 and 500 µg/mL) to evaluate the toxicity of NPs fabricated by PLGA.

## Results

*Morphological analysis*

 The morphology of the NPs prepared by the double-emulsion-solvent evaporation technique was investigated by SEM. The NPs display a uniform spherical shape and smooth surface without any aggregation. There was no morphological difference between pIFN-λ1-loaded NPs (Figure 1a) and empty NPs (Figure 1b). Our results were completely similar to the results reported by J. Samuel *et al*. (22). Our TEM images showed the freeze-drying process and three-month storage period at -70 °C has not changed the morphology of the NPs and no aggregations were visible (Figures 1c and 1d).

*Size, size distribution and zeta potential of the NPs*

 As can be seen from Figure 2, the average particle size of the pIFN-λ1-loaded NPs was around 70 nm larger than empty NPs which was quite rational and correct given the applying of the pIFN-λ1 into F1 formulation. The PdI of both formulation were moderate according to the words of Zetasizer product manager of Malvern Instruments (29). In terms of average PdI and zeta potential, there is no significant difference between F1 and F2 formulation (Table 1).

*Cellular uptake and bioactivity of the NPs*

 As shown in Figure 3b, the pGFP-loaded NPs are engulfed by phagocytic RAW264.7 cells. Four days after treatment, GFP is successfully expressed in this cell line. While transfection of naked pGFP into RAW264.7 cells did not result in expression of GFP using calcium phosphate method. The HEK293T cells failed to uptake the pGFP-loaded NPs, while naked pGFP, which was transfected with calcium phosphate was expressed in this cells after two days (Figure 3d). As can be seen in Table 2, the viability of both cells treated with empty NPs during four days using the trypan blue viability test was more than 90% up to 500 μg/mL.

*Encapsulation efficiency, loading capacity and production yield of pIFN-λ1-loaded NPs*

 The encapsulation efficiency of pIFN-λ1 in NPs was determined using an indirect method with the PicoGreen stain which specifically binds to the dsDNA molecules such as the plasmid. Encapsulation efficiency for the pIFN-λ1-loaded NPs was 75 ± 5% (Table 3). Our result was similar to those researchers who encapsulated DNA in either PLGA (30) or Chitosan (31). In fact, loading capacity obtained in this study stated that there were 8.3 ± 0.6 µg of the pIFN-λ1 per mg of developed PLGA NPs at the end of the manufacturing process. This concept will be more practical for the future pre-clinical and clinical studies than the encapsulation efficiency.

*Release* *pattern* *of pIFN-λ1 from the NPs*

 The *in-vitro* cumulative release profile of pIFN-λ1 from PLGA NPs expressed versus time as a percentage of DNA release of the total DNA that was encapsulated. As can be seen Figure 4, the pattern of DNA release based on the UV absorbance at 260 nm and PicoGreen assay were the same and biphasic. The first phase of release is characterized by a burst release of plasmid from NPs while in the second phase, release converted to the sustained manner until the end of the experiment. The percentage of released plasmid based on the PicoGreen assay was less than 2%, compared to more than 30% based on UV absorbance at 260 nm up to 60 days. 

*Antiviral assay of IFN-λ1 glycoprotein*

 Antiviral activity of IFN-λ1/IL-29 against EMCV has already been proven by Shepperd *et al*., so the purchased recombinant IFN-λ1 used as a positive control in our study. Antiviral activity of IFN-λ1/IL-29 expressed and secreted to the supernatant of HEK293T cells compared to recombinant IFN-λ1 was tested by EMCV challenge in HEP-C2 cells. Our IFN-λ1 (sample called in Figure 5) like the recombinant IFN-λ1 was able to protect HepC-2 cells from viral-induced cytopathic effect when applied 24 h before adding EMCV (Figure 5).

## Discussion

 The advantages of non-viral vectors over viral ones include lower pathogenesis and an easier and more cost-effective method of gene delivery (32). Among the biodegradable polymers used as non-viral vectors, PLGA has been approved by the US FDA and qualifies strongly as a biocompatible polymer for drug delivery, as it is hydrolyzed to glycolic and lactic acid monomers, both of which are easily degraded in the body (33). To date, several techniques such as spray drying (34), cryopreparation (35), and double-emulsion-solvent evaporation (36) have been used by researchers to encapsulate pcDNA in PLGA NPs. In this study, the most common method of pcDNA encapsulation, namely double-emulsion-solvent evaporation was used.

 In our opinion, the first critical parameter in this method was the selection of PLGA of appropriate molecular weight (MW). It has been proven that encapsulation of pcDNA using low-MW polymers results in low encapsulation efficiency (22, 30). Therefore, when we began to design a formulation for the encapsulation of pIFN-λ1 with PLGA, we excluded these polymers. Given that one of our aims was to reduce the cost of pIFN-λ1 encapsulation, a MW of 54–69 kDa was preferred because of its very low cost compared with other high-MW PLGAs (37). 

 Another important factor was the potential solubility of PLGA in common organic solvents such as chloroform and dichloromethane, ethyl acetate, acetone, and others. As the solubility of PLGA depends on the percentage of glycolic acid units present in its structure and decreases with increasing glycolic acid units (38), a lactide:glycolide ratio of 50:50 for PLGA were applied in our formulation to avoid the technical disadvantages of insolubility of this polymer.

 Koby *et al.* in 2007 demonstrated that an increase in the ratio of pcDNA to PLGA from 1:100 to about 1:66 w/w did not have an effect on encapsulation efficiency (30). In addition, using less DNA in the formulation would lower the cost of manufacturing, so we decided to use a ratio of 1:100 w/w in our formulation. By using this ratio, we obtained an encapsulation efficiency of 75 ± 5%. It is necessary to mention that the ratio used in our study (1:100 w/w) was much lower than that used in Vaxfectin-formulated pcDNA (1:1w/w) by Shlapobersky *et al.* in 2012 (39).

 Another important parameter was the emulsifying agent. Polyvinyl alcohol (PVA), the most common emulsifier, is used in emulsion-solvent evaporation methods to stabilize the emulsion because it generates particles of uniform size (40). However, a portion of the PVA will always remain attached to PLGA NPs formulated by this technique due to its interconnection with the polymer. This residual PVA significantly affects the intracellular uptake of NPs. When the amount of PVA residues is increased, the particle becomes more hydrophilic and, as a result, uptake by cells is reduced (41). On the other hand, the use of chloroform instead of acetone and dichloromethane as an organic phase leads to the reduction of residual PVA on the particle surface (41). So, we used not only chloroform but also the minimal amount of PVA (1% w/v) in the encapsulation process to develop uniform NPs that are easily taken up by phagocytic cell lines like RAW264.7. Although only a very small percentage of PVA was used in our formulation, it seems that a fraction of the residual PVA from the surface of the PLGA NPs was still separated and entered the supernatant during the release process. As the maximum absorbance wavelength of the aqueous solution of PVA is the same as that of pcDNA at 260 nm (42), these separated PVAs may have led to interference in the UV-spectrophotometric vitro release assay. So, in our opinion, the results of release measurement by spectrophotometry were not reliable. As our preliminary study demonstrated no interference of PVA in the quantification of pIFN-λ1 using PicoGreen stain (data not shown), the actual release of pIFN-λ1 from the developed NPs was calculated by using PicoGreen in the current study.

 Examination of the results of cellular uptake of pGFP-loaded NPs revealed three important points in our study. First, it showed that the probable mechanism of NP uptake by RAW264.7 was phagocytosis. The uptake of pGFP-loaded NPs occurred in phagocytic RAW264.7 cells but not in HEK293T cells, which are inherently non-phagocytic cells. Second, the integrity of the entrapped pcDNA in PLGA NPs was maintained during the encapsulation process by the double-emulsion-solvent evaporation method. Third, pGFP was successfully released from the NPs into the cytoplasm, then entered the nucleus, and was subsequently transcribed and translated. Walter *et al.* in 2001 also demonstrated that microspheres prepared by spray-drying from different PLGA-type polymers were efﬁciently phagocytosed by primary human macrophages and dendritic cells, but they were not able to monitor GFP expression in these phagocytic cells (34).

 Although the lack of toxicity of PLGA has been confirmed by the USA FDA, some researchers have shown evidence of acidification within the cell after PLGA degradation (43); therefore, the percentage viability of both cell lines was measured at three concentrations, and no toxicity was observed in the tested cells.

 It is already known that cell membranes have large negatively charged domains, which should repel negatively charged nanoparticles (44). Therefore, some researchers apply additives such as chitosan (45), Cetyl Trimethyl Ammonium Bromide (CTAB) (46) , and Dimethyl Dioctadecyl Ammonium Bromide DDAB (47) to their NP preparation process to modify the surface charge or hydrophobicity in order to improve the cellular uptake of polymeric NPs. In this study, without the addition of additives, NPs with a mean zeta potential of −3.3 mV with a standard deviation of ±7 mV were produced. Although these NPs were negatively charged, they were efficiently engulphed by RAW264.7 macrophages. Wilhelm *et al.* in 2003 suggested that the negatively charged particles bind at the cationic sites in the form of clusters because of their repulsive interactions with the large negatively charged domains of the cell surface. The adsorption of the negatively charged particles at the positively charged sites via electrostatic interaction can lead to localized neutralization and a subsequent bending of the membrane, favoring endocytosis for cellular uptake (48).

 The production of an appropriate negative control for NPs produced in the laboratory is required for future animal and clinical studies as well as *in-vitro* ones. Our results showed that there is no difference between IFN-λ1-loaded NPs and empty NPs in terms of PdI and zeta potential, but IFN-λ1-loaded NPs were an average of 70 nm larger than empty NPs. To solve this problem, we suggest that NPs loaded with a backbone of the desired pcDNA instead of empty NPs could be used as a more reliable negative control.

 The freeze-drying (lyophilization) technique is proposed for long-term storage of colloidal NPs, and its success depends on the wise and attentive selection of all components of the NP formulation (49). Our TEM images taken after freeze-drying of pIFN-λ1-loaded NPs showed no aggregation, and this result indicated indirectly the success of our NP formulation.

## Conclusion

 Herein we have presented a simple and cost-effective formulation based on previous literature reports on plasmid encapsulation. No additives were used in this formulation, and the ultracentrifugation step was removed. The frequency of washing steps was decreased. The cheaper PLGA was used to decrease the formulation cost, and the initial plasmid amount was decreased. This formulation yielded non-toxic NPs with suitable physicochemical properties. Therefore, our prepared IFN-λ1-loaded NPs were selected for *in-vitro* expression studies. A comparison of the antiviral properties of pIFN-λ1-loaded NPs with those of naked pIFN-λ1 is ongoing.
